# Reply: PART and amyloid cascade hypotheses are alive and well (but are not so simple)

**DOI:** 10.1007/s00401-025-02921-3

**Published:** 2025-08-06

**Authors:** Heiko Braak, Kelly Del Tredici

**Affiliations:** Clinical Neuroanatomy, Department of Neurology, Center for Biomedical Research, University of Ulm, Helmholtzstraße 8/1, 89081 Ulm, Germany

We concur with Nelson & Crary [19] that all cases with a clinical presentation of Alzheimer’s disease (AD) have abundant filamentous tau inclusions and Aβ deposits in the neocortex [2, 3, 10, 17]. We also understand what PART is supposed to represent, but we disagree with this concept [6, 10, 19]. Greater similarities exist between AD and PART than differences. Thus, the intra-neuronal RNA signatures in hippocampal pyramidal neurons in AD and PART are very similar [26] and the structures of their tau folds are identical [12, 24, 25]. Individuals classified as PART can possess an APOE $\varepsilon 4$ allele [4, 14], which is a major genetic risk factor for late-onset AD [1, 5, 13]. The genotypes $\varepsilon 2/\varepsilon 4$, $\varepsilon 3/\varepsilon 4$, and $\varepsilon 4/\varepsilon 4$ are associated with a significantly increased risk [11, 20]. Three individuals in our study (one NFT stage I, two NFT stage II) were homozygous for APOE $\varepsilon 4$ (Table 2b) [4]. Inasmuch as the $\varepsilon 4/\varepsilon 4$ genotype has been proposed as a form of genetically determined AD [13], it is difficult to imagine them being cases of PART.

The viewpoint that tau inclusions in PART are not on the AD continuum is not confirmed by work done on tau seeding, which has indicated that abnormal tau-dependent propagation (‘prion-like spreading’) in the brain takes place along anatomical connectivities [15, 27] nor is it compatible with the fact that once NFTs develop in the transentorhinal region, nothing can prevent filamentous tau from progressing into the neocortex during NFT stage III, i.e., laterally and posteriorly into areas covering the fusiform and lingual gyri, and beyond (NFT stages IV–VI). The PART hypothesis does not integrate the presence of early axonal tau inclusions within the perforant path in the absence of ${\text{A}}\beta$ deposits, which has implications for the developing disconnection of the neocortex via the entorhinal region from the hippocampal formation that is characteristic of AD [4, 16]. We could not reproduce [9], a previously proposed feature of definite PART [28, 29] as consisting of tau inclusions in hippocampal sector CA2, with the relative sparing of CA1, before the appearance of tau inclusions in the transentorhinal/entorhinal regions; this points again to a greater degree of overlap than difference between PART and AD. Nelson & Crary remark that, ‘in fundamental contradistinction from ADNC, *PART pathology never evolves beyond Braak NFT stage IV’* [19]. If they review our earlier publication [3], this remark and their subsequent statement that ‘there is *never* severe ADNC-type tau pathology without ${\text{A}}\beta$’ must be erroneous because there were seven cases of NFT stage V without ${\text{A}}\beta$ deposits (Table 2). Two of the cases were APOE $\varepsilon 4$ allele carriers.

The authors see a discrepancy between the ‘argument that the pathologic severity of PART is not age-related’ [19] and our Fig. 2 [4], which illustrates that cases without ${\text{A}}\beta$ deposition are not confined to individuals of $\ge 50$ years of age, but that they also occur before the fifth decade. The mean age from NFT stages I–III clearly increases, but in particular, the mean value of 57.4 years for NFT stage I, along with the dispersion of age values, shows that the age range of tau pathology is not confined to older ages. On the contrary, it begins in the twenties (Fig. 2a) [4]. The mention of ‘biological aging’ and ‘universal PART’ [19] does not bring greater clarity. PART contains an artificial cut-off of $\ge 50$  years as a diagnostic criterion; as such, it disregards the existence of tau inclusions in individuals aged $< 50$, including APOE $\varepsilon 4$ carriers (Table 2a in [4]). The reason that PART has been said to increase in frequency and severity with age [19] can probably be attributed to the lack of large autopsy studies on the brains from individuals who died during the first five decades of life (Table 1 in [3], Table 1 in [4]).

The main difference between PART and AD is either the complete absence (phase 0) or the low-level presence (phases 1–2) of ${\text{A}}\beta$ deposition [6]. However, early AD cannot be distinguished from PART with small amounts of ${\text{A}}\beta$ deposits [10, 23]. Moreover, there exists no reason to split the NFT stages into separate groups, such as PART and AD, because *the proposed existence of ‘possible PART’ strongly suggests that earlier and later NFT stages overlap to form a single entity*. NFT stages IV–VI do not develop without stages I–III. The difficulties in separating age-related from AD-related processes are illustrated by Table 2 of the consensus paper [6], where cases with ${\text{A}}\beta$ deposition in the neocortex (phase 1) or in the hippocampus (phase 2), i.e., in cases with NFT/${\text{A}}\beta$ I/1 or III/2, were classified as ‘possible PART’, which is a contradiction in terms and constitutes a potential slippery slope when one considers that the presence of even mild phases of ${\text{A}}\beta$ deposition speaks for an AD-related process rather than a pure tauopathy [10, 29]. According to the amyloid cascade hypothesis, the formation of assembled ${\text{A}}\beta$ in inherited variants of AD is sufficient to cause the process leading to AD. Should one really believe that AD strikes, like a lightening bolt, only when it is clinically diagnosed, usually at NFT stage IV or stages V–VI? If so, therapeutic interventions at that point would be nearly futile. Instead, biomarkers or neuroimaging for recognition of earlier neuropathological disease stages would be preferable for developing and testing causal therapies.

The strongest argument that proponents of the amyloid cascade hypothesis have in their favor is the evidence from the APP-inherited variants of AD [18], and from Down syndrome [8], which indicate that altered APP processing or dysfunction can be primary. In the *APP* mutation cases, abnormal tau inclusions must be downstream of these processes. If *APP* mutations do not induce, but merely promote tau inclusions, then tau filaments must form all the time, perhaps followed by their degradation, because the penetrance of these inherited disease cases is close to 100%. By contrast, mutations in *MAPT*, the tau gene, give rise to abundant tau inclusions, but not to ${\text{A}}\beta$ deposition.

Nelson & Crary emphasize that a “*severe tauopathy in AD/ADNC is downstream of *${\text{A}}\beta$* plaques”* [19]. We can agree with this statement, which would have been inaccurate without the word ‘severe’—better still, ‘severe neocortical’ [22]. By contrast, the amyloid cascade hypothesis posits that ${\text{A}}\beta$ is the first of the two pathological proteins that develop (upstream) and is required for the subsequent development (downstream) of abnormal tau in AD. However, we emphasize that the lesions arise independently, with ${\text{A}}\beta$ deposits initially developing in the neocortex usually a decade later than tau inclusions in the transentorhinal region [3, 22, 23]. It is possible that both abnormal proteins, when present, interact somewhere in the brain, but the exact mechanisms are unknown. In rhesus macaque monkey models of preclinical AD, abnormal 3R + 4R tau inclusions and Aβ plaques arose independently and with spatiotemporal progression patterns similar to those seen in humans, partially recapitulating the patterns of pretangle stages a-c, 1a, 1b, NFT stages III–IV, and phases 0–2 of ${\text{A}}\beta$ deposition [7, 30]. The NFTs consisted of paired helical filaments that resembled those in AD [21] and the macaques were APOE $\varepsilon 4$ homozygotes [30]. They also developed neuritic plaques (NPs) [30]. Notably, pronounced cognitive impairment was assessed in one 38-year-old monkey by means of the delayed non-match-to-sample task of recognition memory [21].

If one were to adhere to the idea that ${\text{A}}\beta$ deposition in the brain is the primary cause of filamentous tau, then cases with abnormal tau arising without ${\text{A}}\beta$ would indeed be outliers and could represent a tauopathy different from AD, such as PART. Nevertheless, the AD-typical tauopathy begins and develops, as a rule, in the absence of ${\text{A}}\beta$ deposition and without clinical symptoms (NFT stages I–III, preclinical phase) [3, 4]. Notably, during these stages, some of the abnormal tau-bearing projection neurons undergo cell death and they do so in the absence of Aβ deposition [4]. The second AD-typical protein, ${\text{A}}\beta$, routinely appears during NFT stages IV–VI, during which clinical disease symptoms gradually become manifest. With the appearance of ${\text{A}}\beta$ plaques, additional and composite lesions form, NPs, consisting of ${\text{A}}\beta$ deposits, dystrophic neuronal processes partly filled with filamentous tau, abnormal astrocytes, and microglial cells. NPs are downstream of ${\text{A}}\beta$ deposition and are not induced by seeding along axonal connectivities. It is obvious that late NFT stages show an increased severity and complexity of the neuropathological and clinical picture in contrast to the previous stages. O*nce again, we see no meaningful rationale for splitting the NFT stages into separate diseases, such as PART and AD; rather, they overlap to form a single entity, in which prodromal AD transitions to full-blown AD*.

In closing, NFT staging remains diagnostically relevant for early- and late-stage abnormal tau inclusions in AD [2]. Ongoing work continues to challenge the PART hypothesis and the universality of the amyloid cascade hypothesis. It remains unproven, in sporadic AD, that abnormal tau is invariably caused by ${\text{A}}\beta$ because filamentous tau can form independently; a possible interaction between ${\text{A}}\beta$ and tau occurs later and the emergence of NPs may be influenced by the presence of Aβ.

## Data Availability

No datasets were generated or analyzed during the current study.

## References

[1] Belloy ME, Napolioni V, Greicius MD (2019) A quarter century of APOE and Alzheimer’s disease: progress to date and the path forward. Neuron 101:820–838. 10.1016/j.neuron.2019.01.056 30844401 10.1016/j.neuron.2019.01.056 PMC6407643

[2] Braak H, Braak E (1991) Neuropathological stageing of Alzheimer-related changes. Acta Neuropathol 82:239–259. 10.1016/0197-4580(95)00021-6 1759558 10.1007/BF00308809

[3] Braak H, Thal DR, Ghebremedhin E, Del Tredici K (2011) Stages of the pathologic process in Alzheimer disease: age categories from 1 to 100 years. J Neuropathol Exp Neurol 70:960–969. 10.1097/NEN.0b013e318232a379 22002422 10.1097/NEN.0b013e318232a379

[4] Braak H, Mayer B, Feldengut S, Schön M, Del Tredici K (2025) Sequence and trajectory of early Alzheimer’s disease-related tau inclusions in the hippocampal formation of cases without amyloid-β deposits. Acta Neuropathol 23(149):50. 10.1007/s00401-025-02862-x 10.1007/s00401-025-02862-x PMC12102137 40407905

[5] Corder EH, Saunders AM, Strittmacher WJ, Schmechel DE, Gaskell PC, Small GW et al (1993) Gene dose of apolipoprotein E and type 4 allele and the risk of Alzheimer’s disease in late-onset families. Science 261:921–923. 10.1126/science.8346443 8346443 10.1126/science.8346443

[6] Crary JF, Trojanowski JQ, Schneider JA, Abisambra JF, Abner EL, Alafuzoff I et al (2014) Primary age-related tauopathy (PART): a common pathology associated with human aging. Acta Neuropathol 128:755–766. 10.1007/s00401-014-1349-0 25348064 10.1007/s00401-014-1349-0 PMC4257842

[7] Datta D, Arnsten AFT (2025) The etiology and prevention of early-stage tau pathology in higher cortical circuits: insights from aging rhesus macaques. Alzheimers Dement 21:e14477. 10.1002/alz.14477 39776253 10.1002/alz.14477 PMC11848412

[8] Davidson YS, Robinson A, Prasher VP, Mann DMA (2018) The age of onset and evolution of Braak tangle stage and Thal amyloid pathology of Alzheimer‘s disease in individuals with Down syndrome. Acta Neuropathol Commun 6:56. 10.1186/s40478-018-0559-4 29973279 10.1186/s40478-018-0559-4 PMC6030772

[9] Del Tredici K, Schön M, Feldengut S, Ghebremedhin E, Kaufman SK, Wiesner D et al (2024) Early CA2 tau inclusions do not distinguish an age-related tauopathy from early Alzheimer’s disease. J Alzheimers Dis 101:1333–1353. 10.3233/JAD-240483 39302368 10.3233/JAD-240483

[10] Duyckaerts C, Braak H, Brion J-P, Buée L, Del Tredici K, Goedert M et al (2015) PART is part of Alzheimer disease. Acta Neuropathol 129:749–756. 10.1007/s00401-015-1390-7 25628035 10.1007/s00401-015-1390-7 PMC4405349

[11] Farrer LA, Cupples LA, Haines JL, Hyman B, Kukull WA, Mayeux R et al (1997) Effects of age, sex, and ethnicity on the association between apolipoprotein E genotype and Alzheimer disease. A meta-analysis. APOE and Alzheimer Disease Meta Analysis Consortium. JAMA 278:1349–1356. 10.1001/jama.1997.03550160069041 9343467

[12] Fitzpatrick AWP, Falcon B, He S, Murzin AG, Murshudov G, Garringer HJ et al (2017) Cryo-EM structures of tau filaments from Alzheimer’s disease. Nature 547(7662):185–190. 10.1038/nature23002 28678775 10.1038/nature23002 PMC5552202

[13] Fortea J, Pegueroles J, Alcolea D, Belbin O, Dols-Icardo O, Vaqué-Alcázar L et al (2024) APOE4 homozygosity represents a distinct genetic form of Alzheimer’s disease. Nat Med 30:1284–1291. 10.1038/s41591-024-02931-w 38710950 10.1038/s41591-024-02931-w PMC13310155

[14] Ghebremedhin E, Schultz C, Braak E, Braak H (1998) High frequency of apolipoprotein E ε4 allele in young individuals with very mild Alzheimer’s disease-related neurofibrillary changes. Exp Neurol 155:152–155. 10.1006/exnr.1998.6860 10.1006/exnr.1998.6860 9743577

[15] Goedert M, Masuda-Suzukake M, Falcon B (2017) Like prions: the propagation of aggregated tau and α-synuclein in neurodegeneration. Brain 140:266–278. 10.1093/brain/aww230 27658420 10.1093/brain/aww230

[16] Hyman BT, Van Hoesen GW, Damasio AR, Barnes CL (1984) Alzheimer’s disease: cell-specific pathology isolates the hippocampal formation. Science 225(4667):1168–1170. 10.1126/science.6474172 6474172 10.1126/science.6474172

[17] Hyman BT, Phelps CH, Beach TG, Bigio EH, Cairns NJ, Carrillo MC et al (2012) National Institute on Aging-Alzheimer’s association guidelines for the neuropathologic assessment of Alzheimer’s disease. Alzheimers Dement 8:1–13. 10.1016/j.jalz.2011.10.007 22265587 10.1016/j.jalz.2011.10.007 PMC3266529

[18] Karran E, De Strooper B (2016) The amyloid cascade hypothesis: are we poised for success or failure? J Neurochem 139(Suppl 2):237–252. 10.1111/jnc.13632 27255958 10.1111/jnc.13632

[19] Nelson PT, Crary JF (2025) PART and amyloid cascade hypotheses are alive and well (but are not so simple). Scientific commentary on: “Sequence and trajectory of early Alzheimer's disease-related tau inclusions in the hippocampal formation of cases without amyloid-beta deposits”. Acta Neuropathol. 10.1007/s00401-025-02920-440767884 10.1007/s00401-025-02920-4

[20] Oveisgharan S, Buchman AS, Yu L et al (2018) *APOE* ε2ε4 genotype, incident AD and MCI, cognitive decline, and AD pathology in older adults. Neurology 90:e2127–e2134. 10.1212/WNL.0000000000005677 29752306 10.1212/WNL.0000000000005677 PMC5996834

[21] Paspalas CD, Carlyle BC, Leslie S, Preuss TM, Crimins JL, Huttner AJ et al (2018) The aged rhesus macaque manifests Braak stage III/IV Alzheimer’s-like pathology. Alzheimers Dement 14:680–691. 10.1016/j.jalz.2017.11.005 29241829 10.1016/j.jalz.2017.11.005 PMC6178089

[22] Price JL, Morris JC (2004) So what if tangles precede plaques? Neurobiol Aging 25:721–723. 10.1016/j.neurobiolaging.2003.12.017 15165694 10.1016/j.neurobiolaging.2003.12.017

[23] Schönheit B, Zarski R, Ohm TG (2004) Spatial and temporal relationships between plaques and tangles in Alzheimer-pathology. Neurobiol Aging 25:697–711. 10.1016/j.neurobiolaging.2003.09.009 15165691 10.1016/j.neurobiolaging.2003.09.009

[24] Shi Y, Murzin AG, Falcon B, Epstein A, Machin J, Tempest P et al (2021) Cryo-EM structures of tau filaments from Alzheimer’s disease with PET ligand APN-1607. Acta Neuropathol 141:697–708. 10.1007/s00401-021-02294-3 33723967 10.1007/s00401-021-02294-3 PMC8043864

[25] Shi Y, Zhang W, Yang Y, Murzin AG, Falcon B, Kotecha A et al (2021) Structure-based classification of tauopathies. Nature 598(7880):359–363. 10.1038/s41586-021-03911-7 34588692 10.1038/s41586-021-03911-7 PMC7611841

[26] Stein-O’Brien GL, Palaganas R, Meyer EM, Redding-Ochoa J, Pletnikova O, Guo H et al (2025) Transcriptional signatures of hippocampal tau pathology in primary age-related tauopathy and Alzheimer’s disease. Cell Rep 44:115422. 10.1016/j.celrep.2025.115422 40085647 10.1016/j.celrep.2025.115422 PMC12019863

[27] Uemura N, Uemura MT, Luk KC, Lee VM, Trojanowski JQ (2020) Cell-to-cell transmission of tau and α-synuclein. Trends Mol Med 26:936–952. 10.1016/j.molmed.2020.03.012 32371172 10.1016/j.molmed.2020.03.012 PMC7529725

[28] Walker JM, Richardson TE, Farrell K, Iida MA, Foong C, Shang P et al (2021) Early selective vulnerability of the CA2 hippocampal subfield in primary age-related tauopathy. J Neuropathol Exp Neurol 80:102–111. 10.1093/jnen/nlaa153 33367843 10.1093/jnen/nlaa153 PMC8453611

[29] Walker JM, Orr ME, Orr TC, Thorn EL, Christie TD, Yakoda RT et al (2024) Spatial proteomics of hippocampal subfield-specific pathology in Alzheimer’s disease and primary age-related tauopathy. Alzheimers Dement 20:783–797. 10.1002/alz.13484 37777848 10.1002/alz.13484 PMC10916977

[30] Zeiss CJ, Huttner A, Nairn AC, Arnsten A, Datta D, Strittmatter SM et al (2025) The neuropathologic basis for translational biomarker development in the macaque model of late-onset Alzheimer’s disease. J Alzheimers Dis 104:1243–1258. 10.1177/13872877251323787 40095666 10.1177/13872877251323787 PMC12380261

